# Inclined Fiber Pullout from a Cementitious Matrix: A Numerical Study

**DOI:** 10.3390/ma9100800

**Published:** 2016-09-26

**Authors:** Hui Zhang, Rena C. Yu

**Affiliations:** 1 College of Civil Engineering & Architecture, Zhejiang University, Hangzhou 310058, China; huizhangzju@zju.edu.cn; 2 ETSI de Caminos, C. y P., University of Castilla-La Mancha, Ciudad Real 13071, Spain

**Keywords:** fiber-reinforced concrete, pullout response, internal friction resistance

## Abstract

It is well known that fibers improve the performance of cementitious composites by acting as bridging ligaments in cracks. Such bridging behavior is often studied through fiber pullout tests. The relation between the pullout force vs. slip end displacement is characteristic of the fiber-matrix interface. However, such a relation varies significantly with the fiber inclination angle. In the current work, we establish a numerical model to simulate the entire pullout process by explicitly representing the fiber, matrix and the interface for arbitrary fiber orientations. Cohesive elements endorsed with mixed-mode fracture capacities are implemented to represent the bond-slip behavior at the interface. Contact elements with Coulomb’s friction are placed at the interface to simulate frictional contact. The bond-slip behavior is first calibrated through pull-out curves for fibers aligned with the loading direction, then validated against experimental results for steel fibers oriented at 30${}^{\circ }$ and 60${}^{\circ }$. Parametric studies are then performed to explore the influences of both material properties (fiber yield strength, matrix tensile strength, interfacial bond) and geometric factors (fiber diameter, embedment length and inclination angle) on the overall pullout behavior, in particular on the maximum pullout load. The proposed methodology provides the necessary pull-out curves for a fiber oriented at a given angle for multi-scale models to study fracture in fiber-reinforced cementitious materials. The novelty lies in its capacity to capture the entire pullout process for a fiber with an arbitrary inclination angle.

## 1. Introduction

Since conventional concrete tends to fail in a brittle manner under excessive loading, fibers are often added to improve its ductility and durability. Typical examples of high-performance fiber-reinforced cement-based composites are slurry infiltrated fiber concrete (SIFCON) [1,2,3], engineered cement composites (ECC) [4,5,6], steel fiber-reinforced self-compacting concrete [7,8] or high performance concrete [9], as well as ultra-high toughness cementitious composites [10]. The effectiveness of the given type of fibers, steel fibers in particular, is often assessed through a pullout test, in which the force required to pull a fiber out of the hardened concrete is measured. The transmission of this force is achieved through the interfacial bond, defined as the shear stress at the interface between the fiber and the surrounding matrix [7,11,12,13]. In order to capture the interface failure, the stress criterion [14,15] or the energy criterion [16,17,18], as well as cohesive approaches where bond stress is determined by the relative slip between the fiber and matrix [12,16,19,20,21] have been adopted.

Naaman et al. [12] pointed out that aligned fibers (i.e., load is applied at the fiber longitudinal direction) exhibit two types of shear bonds at the interface: elastic and frictional. When the elastic shear stress at the interface exceeds the bond strength of the interface, the bond becomes frictional in nature. This means that the bond-broken process was considered abrupt, not gradual. Based on this hypothesis, Naaman et al. [12] developed a fundamental study of the bond in fiber-reinforced cement composites, as well as the relationship between pullout curves and bond shear stress vs. slip curves. In their analysis, a complete pullout curve was analytically derived from an assumed bond-slip relationship, and the dual problem in which the bond-slip relationship was obtained from an experimental pullout curve was solved. Furthermore, they related the frictional behavior of the interface by shrink-fit and fiber-matrix misfit theory. Other examples of aligned fibers can be found in [22,23]. For instance, by focusing on the interfacial properties, Chanvillard [22] developed a micro-mechanical model to account for the different phenomena observed in a non-straight fiber. Ellis et al. [23] conducted the simulation of a single fiber taking into account the fiber morphology. Even though good agreement was obtained, only aligned fibers were considered in these two models.

When fibers are randomly distributed, however, besides bond strength and friction along the interface, additional phenomena, such as fiber bending, matrix spalling and local friction effects, are involved [24,25,26,27]. Furthermore, the contributions of these micro-mechanisms are dependent on the fiber inclination angle and the fiber material properties [13,18,24,25,26,28,29]. The contribution of fiber bending increases with inclination angle. The fiber curvature also plays an important role in the distribution of pressure against the surrounding matrix. Due to the local curvature of the fiber and residual stress at the interface, the matrix tends to crack and spall [24,26,29]. This has a direct impact on the effective embedment length and deformation within the fiber. Moreover, snubbing friction near the fiber exit point can increase the pull-out resistance [24]. Considerable efforts have been put into modeling the mentioned phenomena involved in the pullout process of inclined fibers. Some models considered the bending mechanism of inclined fibers while ignoring the matrix spalling. For example, Mortons and Groves [30] employed an elementary beam theory to calculate the force needed to produce a plastic hinge in the fiber as it is withdrawn from the matrix. In this way, the magnitude of the additional pull-out force due to fiber inclination was accounted for. The model reproduced well the experimental observations for lower inclination angles, but failed to do so for steeper ones. Li et al. [24] assumed an exponential increase of the pullout load with fiber orientation to capture the maximum pullout load; however, detailed information of the entire pullout process was omitted. By treating the fiber as a beam on an elastic foundation with variable stiffness and the possibility of spalling, Leung and Li [18] developed a micro-mechanical model to analyze the coupled fiber bending-matrix spalling mechanism and to determine crack bridging stress in random fiber-reinforced brittle matrix composites. Afterwards, the model was extended to ductile fibers [31]. Nevertheless, friction was left out of the picture. More comprehensive models, like that of Cailleux et al. [29] and Fantilli et al. [20], involve considerable parameters, which can only be estimated through pullout tests of aligned, as well as inclined fibers. In addition, tedious numerical iterative procedures are required.

In spite of the above models, detailed simulations to consider all of the aforementioned phenomena have been seldom conducted to explicitly model the entire pullout process for fibers at an arbitrary angle and considering both fiber bending and matrix spalling. In the current work, we endeavor to do so. Cohesive models capable of representing mixed-mode fracture and Coulomb’s friction are employed to simulate the interface between fiber and matrix: both the de-bonding and frictional phases. In addition, fiber bending and matrix spalling are taken into account naturally thanks to the explicit representation of the fiber, the matrix and the interface in between. The rest of the paper is organized as follows. The interface bond characteristics and matrix spalling are presented in Section 2. Model calibration and validation are given in Section 3. Numerical results and parametric studies are depicted in Section 4 and Section 5. Finally, relevant conclusions are drawn in Section 6.

## 2. Bond Characterization and Matrix Spalling

In this section, two essential features that determine the pullout response of a fiber with arbitrary orientation are explained in detail; namely bond characterization and the quantification of matrix spalling. The former deals with the identification of the distinct mechanisms behind interface bond deterioration and friction from pullout tests of both aligned and inclined fibers. The latter tackles the matrix failure near the fiber exit point when an inclined fiber is pulled out.

### 2.1. Interface Bond Characterization

The shear stress vs. slip relationship is considered to be a constitutive property of the interface. Its characterization is of primary importance for predicting both the mechanical and fracture properties of fiber-reinforced composites. Naaman et al. [11] attributed the presence and combination of distinct components to the complex nature of the bond: physical and chemical adhesion, the mechanical effect of deformed or hooked fibers, the entanglement of fibers and friction, which is greatly influenced by confinement. In the current work, we concentrate on the case of a single smooth straight fiber being pulled out from a cementitious matrix. Consequently, only the interface adhesion and friction are dealt with. By fitting with the experimental data of Leung and Shapiro [26], the contribution of internal friction to interface strength will be identified and quantified. It needs to be emphasized that the same methodology can be applied for hooked or deformed steel fibers with additional efforts to represent the detailed fiber geometry.

Either for an aligned fiber or an inclined one, there exists certain frictional resistance after debonding when being pulled out. This friction is mainly dominated by the surface roughness, and it is often assumed to be constant regardless of the fiber orientation. This component is defined as internal friction, $\tau_{f}\left(s\right)$, which is a function of the slip displacement, *s*. As for inclined fibers, however, the mechanisms are quite different. Pullout load can be decomposed into a parallel force and a perpendicular one. The former pulls the fiber out, while the latter bends the fiber and changes its direction during the pullout process. In addition, due to the non-uniform compression at the interface, the brittle matrix may spall, and the fiber embedment length is reduced.

**Figure 1:**
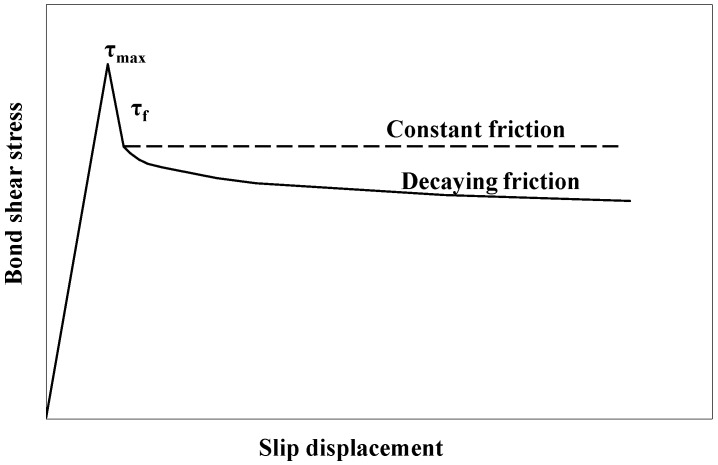
Bond stress vs. relative slip at the interface: the contribution of friction.

In order to take into consideration the above phenomena, we propose a generic constitutive law that has three constituents to govern the interface deterioration process as follows:

(1) $$\tau \left(\theta ,s\right)=\tau_{b}\left(s\right)+\tau_{f}\left(s\right)+\mu \;p\left(\theta \right)$$

where *μ* is Coulomb’s friction coefficient, *θ* is the fiber inclination angle with respect to the external load direction, whereas p(θ) is the compressive stress as a function of *θ*. The first term, $\tau_{b}\left(s\right)$, illustrates the interfacial bond relation due to internal physical and chemical cohesion; it is often a decaying function of the slip displacement, *s*. In addition, $\tau_{b}\left(s\right)$ is cohesive in nature; thus, it can be modeled through cohesive elements [32,33] with Mode-II fracture capacity. The second term, $\tau_{f}\left(s\right)$, is a contribution from internal friction, which resists the motion between the fiber and the matrix; it can be constant or decreasing; see Figure 1. The third term gives the shear stress due to dry friction, which plays a role only when the fiber is oriented at a non-zero angle with respect to the load direction.

In the current work, the bond stress is assumed to be linear-decreasing with respect to *s*, whereas internal friction is assumed to be constant; namely:

(2) $$\begin{array}{c} \tau_{b}\left(s\right)=\left(\tau_{max}-\tau_{f_{c}}\right)\left(1-\frac{s}{s_{b_{c}}}\right),\;0\leq s\leq s_{b_{c}} \end{array}$$

(3) $$\begin{array}{c} \tau_{f}\left(s\right)=\tau_{f_{c}}\; \end{array}$$

where $\tau_{max}$ is the maximum shear stress resisted at the interface; it covers the effect of both the internal bond and internal friction. $s_{b_{c}}$ is the critical slip displacement, when the bond is completely broken. $\tau_{f_{c}}$ is the internal frictional resistance, which is a property of the interface.

The constitutive law encompassed in Equations (2) and (3) highlights a gradual failure at the interface; see Figure 1. This, on the one hand, differs from the abrupt bond failure assumed by Naanman et al. [12]; on the other hand, it appears particularly important, since in most practical fiber applications, the slip displacement is less than 1 mm; see Yu et al. [34]. When working in a corrosive environment, the maximum allowed crack opening in steel fiber-reinforced composites is around 0.3 mm. This makes it especially important to be able to capture the detailed failure process at small slip values.

### 2.2. Matrix Spalling

During the pullout process of inclined fibers, due to the local curvature and stretching of the fiber segment at the free end, matrix spalling is inevitable [24,26,29]. Detailed modeling of the spalling process is not an easy task. Since the deterioration process occurs within a narrow band near the interface, excessive mesh distortion often leads to convergence problems and, thus, impedes the further modeling of the entire pullout process. Consequently, simplifications are often assumed, so that the spalling part is taken off from the numerical representation, once the matrix tensile strength is reached [27,29,35,36]. The length of the eroded matrix along the fiber direction is the so-called spalling length, denoted as $L_{sp}$ by Laranjeira et al. [35]. A simplified failure criterion was adopted in [35], i.e., if the spalling force imposed by fiber curvature is larger than the resistance provided by the matrix at the cracked surface, the matrix will spall. Accordingly, an estimated length of the spalled matrix can be obtained as follows:

(4) $$a\;L_{sp}^{2}+b\;L_{sp}+c=0$$

where:

(5) $$a=\frac{\sqrt{2}}{\sin\theta }+\frac{\cos\theta }{\sin^{2}\theta },\;b=\frac{d_{f}}{\sin\theta },\;c=-\frac{P_{max}\;\sin\theta }{f_{t}}.$$

In the above expressions, $d_{f}$ stands for the fiber diameter, *θ* is the inclination angle, $P_{max}$ is the peak pullout load of an aligned fiber, whereas $f_{t}$ is the tensile strength of the matrix. The estimated values from Equation (4) closely match the ones measured through scanning electron microscopy (SEM) by Leung and Shapiro [26].

In the current work, Equation (4) is adopted as a first approximation to represent the matrix wedge, which is to be spalled off later on. A line of cohesive elements connects the wedge to the main part of the matrix. Trial runs are performed to determine the moment at which the matrix wedge should be deactivated. From then on, the elements within the matrix wedge cease to contribute to the overall stiffness. It bears emphasis that such a treatment is only to save computational time without detriment to the modeled phenomenon, since the first principal stresses within the matrix are checked, and the spalled length is adjusted if necessary, to ensure that the matrix would not present hyper-strength.

## 3. Finite Element Methodology: Model Calibration and Validation

**Figure 2:**
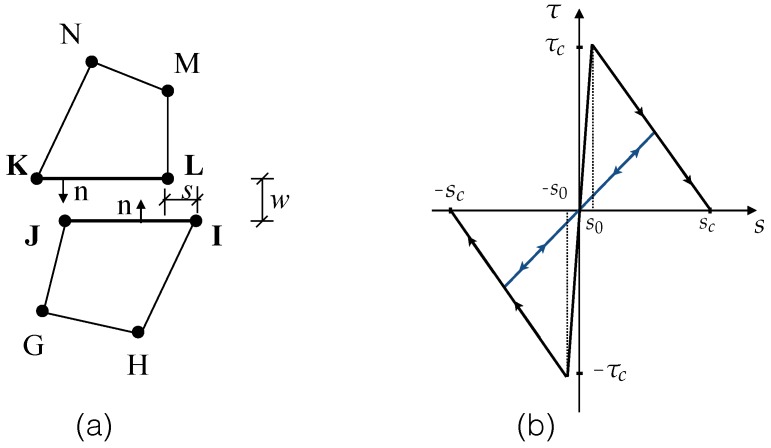
(**a**) A 2D interface element I-J-K-L formed between two four-node solid elements G-H-I-J and K-L-M-N, where *s* and *w* are the tangential and normal displacement jumps, and *n* is the normal direction of the two line segments; (**b**) the bond-slip law (shown for a linear-decreasing case).

As mentioned before, the objective is to explicitly model the matrix, the fiber and the interface in between. In this section, a 2D setting, as shown in Figure 2a, is chosen. Both the fiber and the matrix are represented with four-node iso-parametric elements (K-L-M-N and G-H-I-J), whereas the interface is discretized into pairs of two-node line segments (I-J and K-L). The constitutive behavior of the interface is governed by a cohesive law, as shown in Figure 2b. The initial ascending part is aimed at eliminating the numerical instability, which can be caused by near-zero slip displacements derived from machine error. Consequently, the zero-damage is set for a small slip displacement, $s_{0}$, when the bond strength, $\tau_{c}$, is attained; whereas the complete damage is reached for the critical slip displacement, $s_{c}$, beyond which, only frictional forces exist at the interface. Additionally implemented is a pair of contact-target elements (I-J and K-L) superposed at the same location as the interface elements, so that when the interface is under compression, no penetration should occur.

The above configuration is implemented in the commercial software ANSYS 12.0, using its APDL programming language. Regarding the constitutive laws, linear elasticity for the matrix and bilinear plasticity with isotropic hardening for steel fiber are adopted, respectively.

### 3.1. Experimental Setup of Leung and Shapiro, 1999

In order to assess the effect of fiber yield strength on reinforcement efficiency in terms of maximum crack bridging force and total energy absorption, Leung and Shapiro [26] carried out pullout tests for steel fibers of different yield strengths and inclination angles at 0${}^{\circ }$, 30${}^{\circ }$ or 60${}^{\circ }$. All of the fibers are 0.5 mm in diameter and 22 mm in length. The pullout specimens are blocks of 25.4 mm× 12.7 mm × 9.5 mm in size, with an effective fiber length (equal to the embedment length in this case), $L_{f}$, of 10 mm. The material parameters for the matrix, the fiber and the interface are given in Table 1, whereas the yield and tensile strengths of the four types of fibers are listed in Table 2. Additionally listed in Table 2 is the critical fiber length, i.e., the maximum embedded length for a fiber to be pulled out from a matrix without rupture [24]. It is related to the maximum shear stress, $\tau_{max}$, fiber tensile strength, $f_{r}$, fiber cross-section, $A_{f}$, and perimeter, $p_{f}$, as follows

(6) $$L_{c}=\frac{A_{f}\;f_{r}}{p_{f}\;\tau_{max}}=\frac{\left(\pi \;d_{f}^{2}/4\right)\;f_{r}}{\pi \;d_{f}\;\tau_{max}}=\frac{d_{f}\;f_{r}}{4\;\tau_{max}}.$$

It needs to be pointed out that this estimation is for aligned fibers only; in the case of inclined ones, this length is considerably smaller due to the contribution of fiber bending.

### 3.2. Identification of the Fiber-Matrix Interface Properties

In order to extract the interface properties from experimental pullout curves, we first introduce the concept of apparent interfacial shear stress, which is defined as:

(7) $$\tau^{*}=\frac{1}{L_{e}}\int_{0}^{L_{e}}\tau (s)ds$$

where τ(s) is the actual bond stress distribution at the interface and $L_{e}$ is the initial embedment length, the value of which is equal to $L_{f}$.

Assuming the fiber-matrix interface property is uniform, the maximum pullout load and peak frictional load are respectively calculated as:

(8) $$P_{max}=\pi \;d_{f}\;L_{e}\;\tau_{max},\;\;P_{f}=\pi \;d_{f}\;L_{e}\;\tau_{f_{c}}.$$

**Figure 3:**
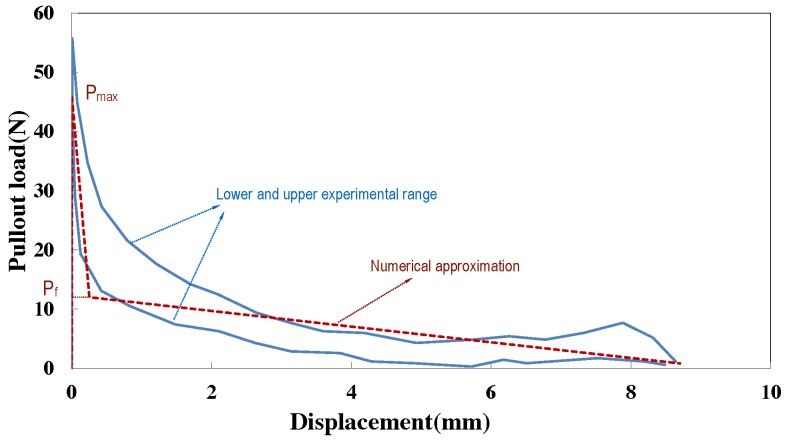
Experimental range (light blue lines) for pullout curves for aligned fiber Type 2 (yield strength 469 MPa) [26] and its bi-linear numerical approximation (red dotted line). $P_{max}$ and $P_{f}$ are the peak and the transitional pullout forces.

The values for $P_{max}$ and $P_{f}$ are determined from the pullout response of aligned fibers, as shown in Figure 3. Note that the values for $P_{max}$ and $P_{f}$ are averaged for the four types of fibers listed in Table 2 to obtain those of $\tau_{max}$ and $\tau_{f_{c}}$, as well as their standard deviations in Table 3. The critical slip for interfacial bond, $s_{b_{c}}$, 0.3 mm, is determined through trial and error, so that the first decaying branch of the numerical pullout responses, as demonstrated in Figure 3, should fall within the experimental range. As regards the friction coefficient given in Table 3, it is estimated according to the experimental results of Chanvillard [22], which was also adopted by Laranjeira et al. [27].

### 3.3. Numerical Model

The in-plane dimensions and boundary conditions to simulate the pullout tests performed by Leung and Shapiro [26] are illustrated in Figure 3. Note that within a two-dimensional plain stress framework, the fiber thickness, $T_{f}$, is calculated through Equation (9) so that the contact area at the interface is the same as that of the original one. In the same way, the fiber height, $H_{f}$, is determined via Equation (10) so that the moment of inertia of the original fiber is the same. For the case of a fiber diameter of 0.5 mm, $T_{f}$ and $H_{f}$ are computed as 0.785 and 0.36 mm, respectively.

(9) $$\begin{array}{ccc} T_{f} & = & \frac{\pi \;d_{f}\;L_{f}}{2\;L_{f}} \end{array}$$

(10) $$\begin{array}{ccc} H_{f} & = & {\left(\frac{\pi \;d_{f}^{4}/64}{T_{f}/12}\right)}^{1/3} \end{array}$$

**Figure 4:**
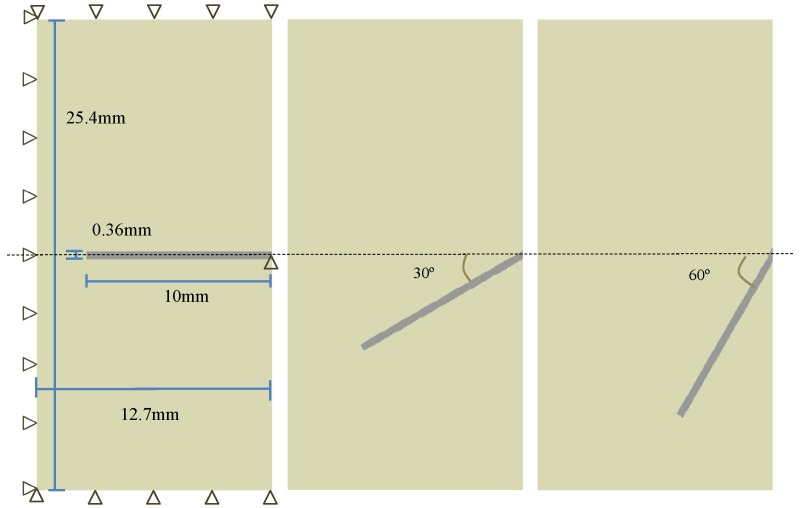
In-plane dimensions and boundary conditions (the same for all cases, shown only for aligned fibers) for the pullout tests performed by Leung and Shapiro [26], with fiber inclination angles of 0${}^{\circ }$, 30${}^{\circ }$ and 60${}^{\circ }$.

Additional described in Figure 4 are the boundary conditions imposed. Vertical displacements are prevented on the top and bottom sides, whereas horizontal movements are impeded on the left. The right end of the fiber is fixed in the vertical direction so that only horizontal movement is permitted. Note that the same boundary conditions are imposed for inclined fibers. The pulling process is carried out with intervals of 0.001 mm in the horizontal direction until 0.3 mm, followed with increments of 0.01 mm until the end.

### 3.4. Mesh Description

**Figure 5:**
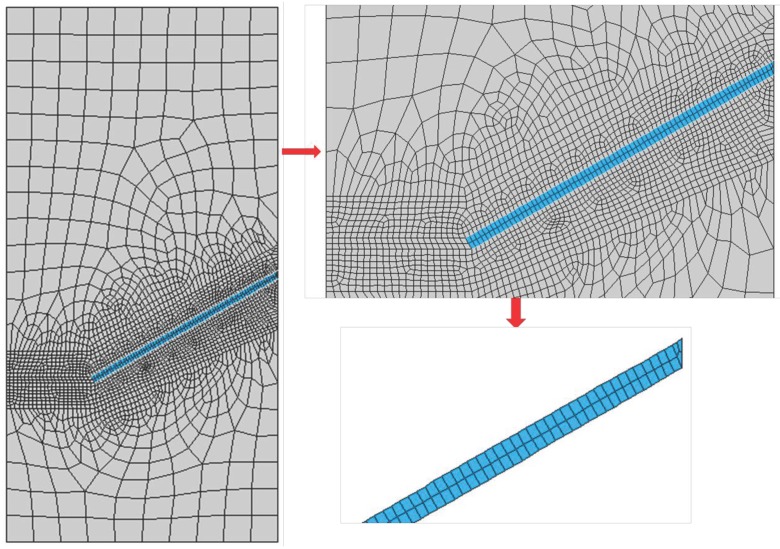
Typical mesh (**left**), zoomed in around the fiber (**top right**) and discretisation of the fiber (**bottom right**).

A typical mesh and detailed element distribution around the fiber for the inclination angle of 30${}^{\circ }$ are demonstrated in Figure 5. Note that the right end of the fiber leans on a matrix wedge, which will spall later on. For this particular case, the matrix and the fiber consist of 2198 and 154 four-node solid elements, respectively, whereas 289 two-node contact pairs are placed at the interface. The mesh sensitivity analysis performed to achieve a balance between the computational efficiency and accuracy is going to be presented in Section 4.

## 4. Numerical Results and Discussion

In this section, mesh sensitivity analysis is first conducted along the fiber transverse and longitudinal directions in order to determine the particular mesh to be employed for further studies. Second, the entire pullout load vs. slip displacement curves are extracted to compare with those obtained experimentally by Leung and Shapiro [26]. Third, the von Mises stress and the first principal stress evolutions are explored both for the fiber and the matrix. Finally, the pullout work is obtained.

### 4.1. Mesh Sensitivity Analysis

**Figure 6:**
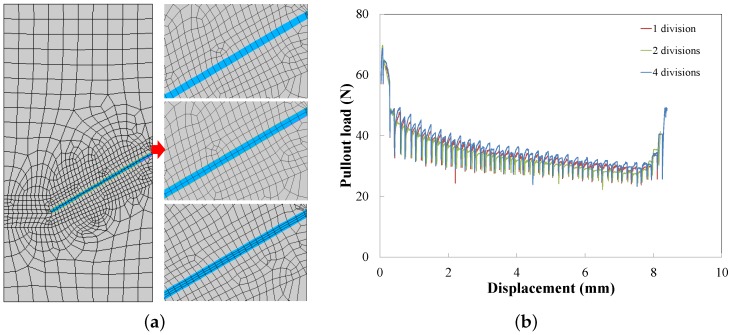
(**a**) Different numbers of divisions in the fiber transverse direction (one, two and four); and (**b**) the corresponding pullout load vs. displacement responses.

The mesh sensitivity analysis is carried out for the inclination angle of 30${}^{\circ }$ and the yield strength of 635 MPa (Type 3 in Table 2). Two kinds of mesh sensitivities are studied: the refinement in the transverse direction and along the axial direction of the fiber. The former is to check the capacity of the mesh in the fiber to bear bending moments, whereas the latter is to assess if the discretisation is fine enough to resolve the slip length. In the transverse direction, the fiber is split into one, two or four divisions (see Figure 6a); the corresponding load-displacement curves are plotted in Figure 6b. On the one hand, convergence is achieved as the mesh is refined. On the other hand, slight differences among the three curves are perceivable. This means that the mesh sensitivity in the transversal direction is rather modest. Meshes of two divisions across the transverse direction are employed for further studies.

**Figure 7:**
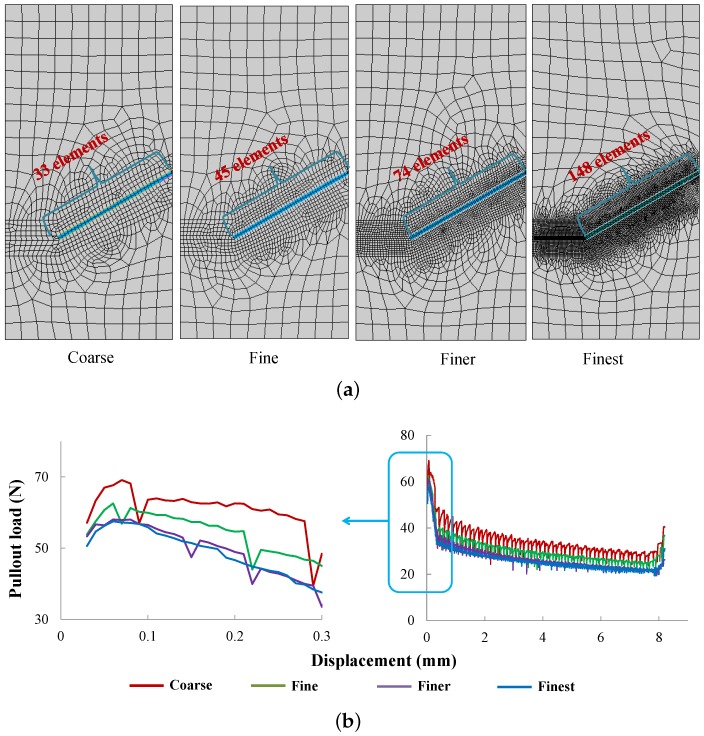
(**a**) Different element sizes (number of divisions) along the fiber; and (**b**) the corresponding pullout load-displacement responses.

Along the longitudinal direction of the fiber, four different element sizes are considered, 0.303 mm, 0.222 mm, 0.135 mm and 0.068 mm, which lead to 33, 45, 74 and 148 divisions along the 10-mm length (see Figure 7a); the corresponding pullout load vs. slip end displacement curves are depicted in Figure 7b. Note that as the mesh gets finer, the response is smoother, and convergence is achieved. Specifically, the same peak load is obtained for the two finer meshes. In order to keep a balance between the computational efficiency and accuracy of the results sought, the mesh size of 0.135 mm is selected for further studies. It needs to be emphasized that the mesh sensitivity in the longitudinal direction is more pronounced than that in the transverse direction. In addition, from Figure 7b, it is observed that the maximum pullout load was achieved at a slip displacement of 0.07 mm, which agrees with the statement of Morton and Groves [30], who claimed that this value should be of the order of, but less than half, a fiber diameter.

### 4.2. Validation against Experimental Pullout Load vs. Displacement Response

**Figure 8:**
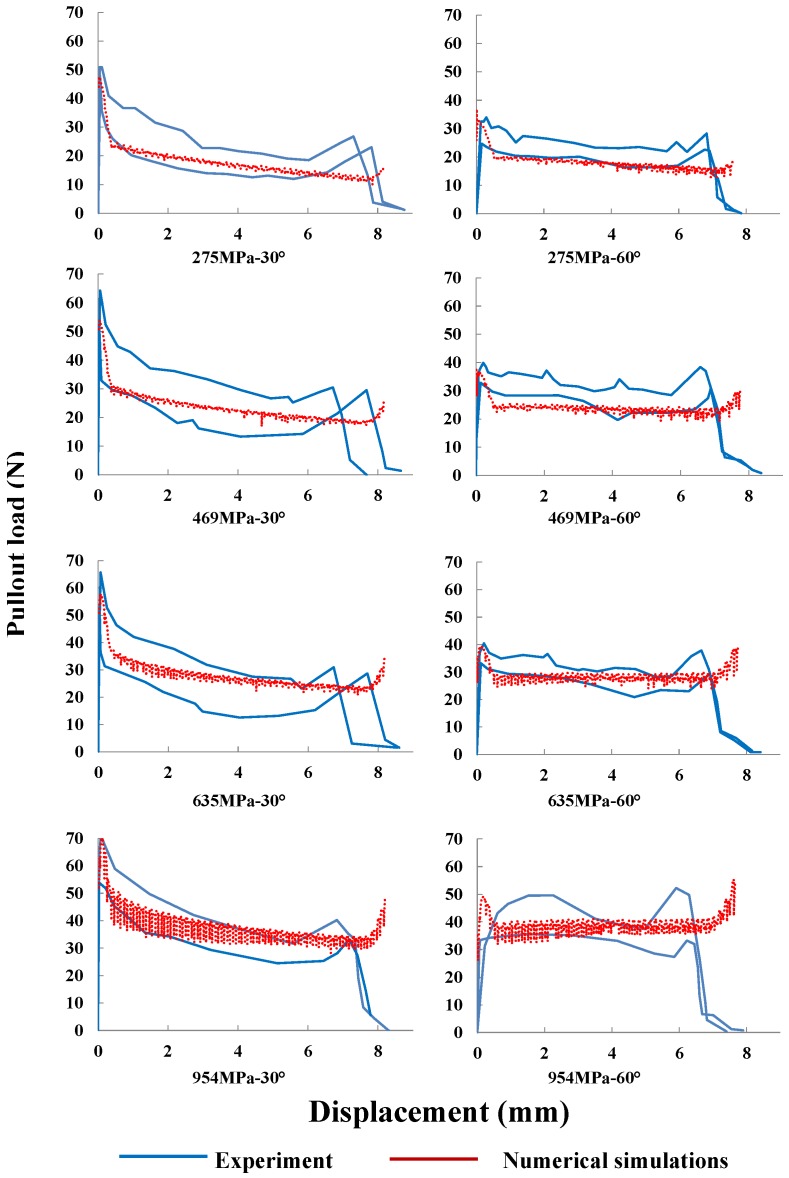
Numerical-experimental [26] comparison: complete pullout curves for the four yield strengths given in Table 2; the fiber is inclined either at 30${}^{\circ }$ (**left** column) or 60${}^{\circ }$ (**right** column).

In order to verify the previously developed methodology, we compare the entire pullout curves with their experimental counterparts given by Leung and Shapiro [26]. This comparison is displayed in Figure 8 for fibers inclined at 30${}^{\circ }$ and 60${}^{\circ }$ with four different yield strengths given in Table 2. Note that both the peak loads and the general tendency are well captured; the numerical curves fall within the experimental range; in particular, the rising tail at the end of each pullout process is also reproduced.

It needs to be pointed out that as the fiber yield strength increases, the numerical pullout curves exhibit more oscillations. This means that the relative slip between fiber and matrix is more unstable; thus, finer meshes are needed to reduce the noise observed in those curves for fibers of higher strength.

### 4.3. Stress Evolution within the Fiber

**Figure 9:**
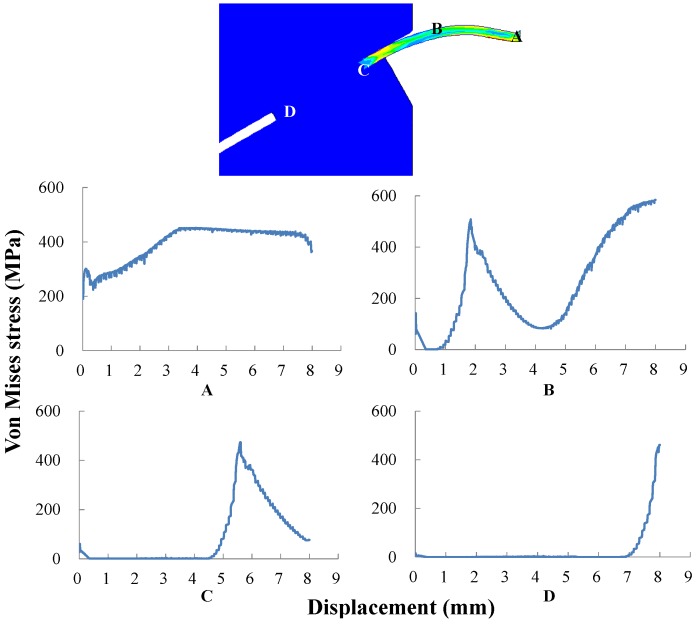
Four positions (**A**, **B**, **C** and **D**) within the fiber during pullout and the corresponding von Mises stress evolutions for Type 2 fibers (fiber yield strength 469 MPa).

Taking Type 2 fiber (yield strength 469 MPa) with an inclination angle of 30${}^{\circ }$ as an example, the von Mises stress evolutions for several characteristic points within the fiber are examined. These points are the pullout end A, the embedded end D and two intermediate ones B (location of the matrix spalling) and C, as depicted in Figure 9. Note that at Point A, the first peak stress was obtained when the pullout load reached its maximum due to interface debonding. Then, after a slight decrease, this stress increased again until yielding at the slip end displacement of 3 mm. Similar peaks are observed for B and C at slip displacement of 0.3 mm and a second peak upon yielding at 2 mm for Point B and 5 mm for Point C, respectively. The second peak is attributed to the stress concentration due to the cusp formed by matrix spalling. This is the snubbing effect described by Li et al. [24].

**Figure 10:**
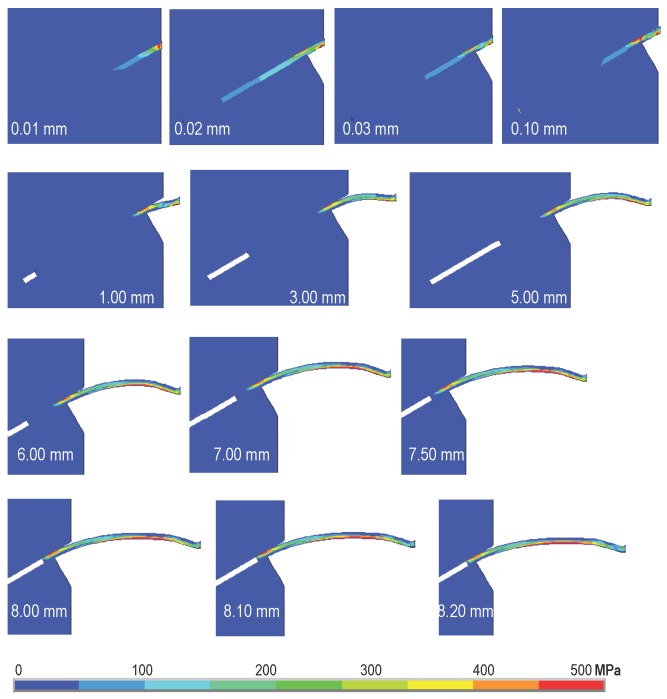
The first principal stress evolution for pullout displacement from 0.01 mm to 8.20 mm for a fiber inclination of 30${}^{\circ }$ and a yield strength of 469 MPa (Type 2 in Table 2).

**Figure 11:**
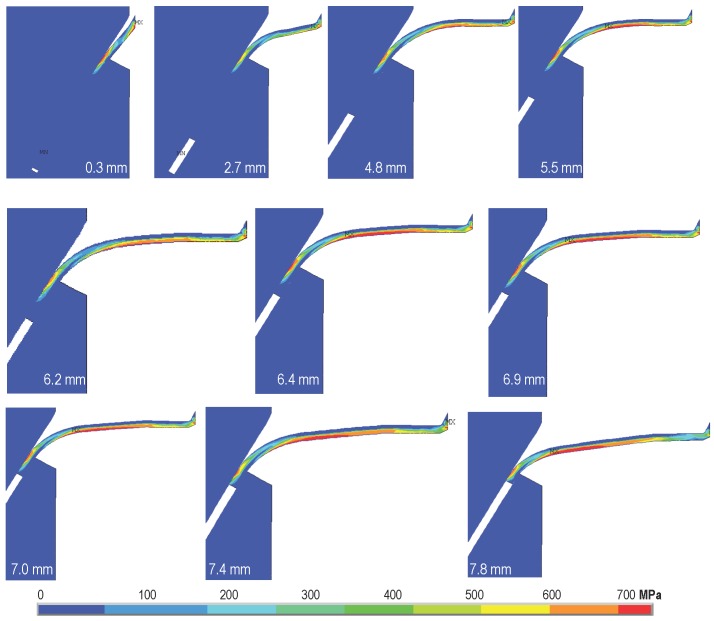
The first principal stress evolution for pullout displacement from 0.3 mm to 7.8 mm for a fiber inclination of 60${}^{\circ }$ and a yield strength of 635 MPa (Type 3 in Table 2).

In Figure 10 and Figure 11, the first principal stress distributions in the fiber at different loading stages are plotted for Type 2 fiber inclined at 30${}^{\circ }$ and Type 3 fiber inclined at 60${}^{\circ }$, respectively. Note that during the pullout process, there are stress gradients both in the transversal direction and along the longitudinal one, which indicates a clear bending contribution. Stress concentration is also observed at the fiber exit point. In addition, the maximum tensile stress is always inferior to the fiber tensile strength. This means that the fiber was pulled out, but not broken. Furthermore, increasing separation is observed at the upper interface as the pullout process advances; this requires a numerical model that is able to capture a mixed-mode interface failure to correctly reproduce the experimentally-observed phenomenon.

There is an interesting result that goes against our intuition, which is that the stress along the fiber should be compressive on the concave surface and tensile on the convex one. However, the opposite is observed particularly in Figure 11. This is because a fiber segment bends the most right before passing the cusp; then, it is straightened by the pulling force, leading to compression on the convex part and tension on the concave part. Additionally observed from Figure 11 is that the length of the red region in the fiber is almost constant since the segment on the right gets unloaded as the left part is being stressed. This further demonstrates the stable deterioration of the interface during pullout. Only when a small fragment of the fiber is remaining in the matrix block, it serves as a hook, which grips the matrix tightly due to its relatively large stiffness. This leads to the tail-rise phenomenon, which is increasingly obvious when the yield strength is larger, since a stronger grip is formed.

### 4.4. Stress Evolution in the Matrix

**Figure 12:**
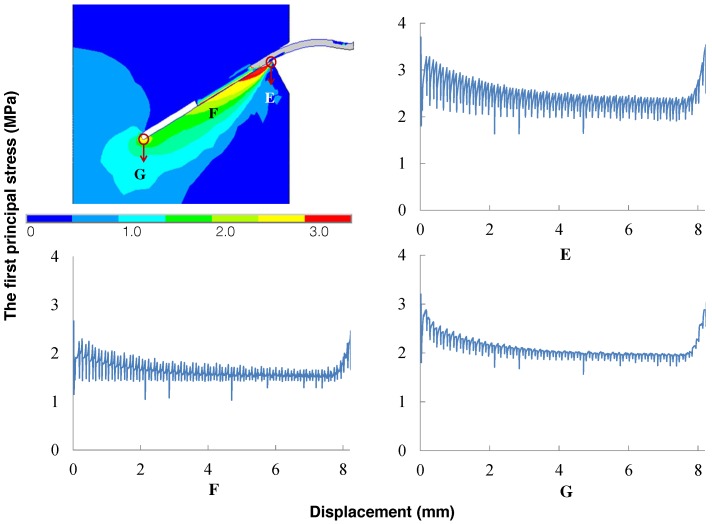
Three positions (**E**, **F** and **G**) in the matrix and the corresponding first principal stress evolutions during the pullout process.

For the matrix, we are more concerned with the tensile stress distribution to ensure that no fracture should take place where matrix spalling is not expected. Three representative points, E, F and G (see Figure 12), are selected to display the first principal stress evolution in the matrix. Point E is where the matrix is expected to spall. Point G is the location where the fiber is anchored, whereas F is the point in the matrix close to the fiber center. Since the tensile strength was not measured, we estimate it to be 1/12 of the compressive strength, which is 3.0 MPa, as shown in Table 1. Note that at Point E, there is relatively large stress oscillation during the pullout process. This indicates, on the one hand, the stressing-relaxing cycle endured by the matrix. On the other hand, it can be attributed to the fact that the spalled matrix is assigned with a zero stiffness at the moment of spalling, whereas the real failure process is gradual. The stress evolution curves at Points F and G, assimilate those of global pullout curves in Figure 8, each with a different amplitude. Furthermore, it is noted from Figure 12 that the maximum tensile stress due to the axial pullout of the fiber and bending load occurs at the close region of the fiber exit point. This agrees with the assumption adopted by Zhang and Li [37] in their study on the effect of inclination angle on fiber rupture load in fiber-reinforced cementitious composites.

### 4.5. Variation of the Pullout Work with Respect to Fiber Yield Strength

**Figure 13:**
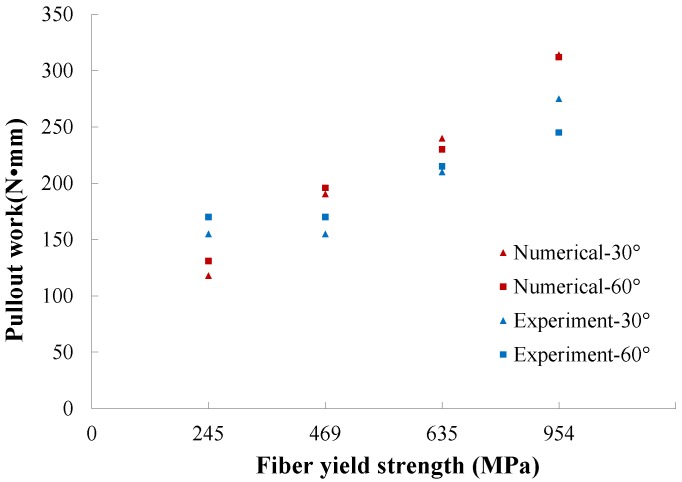
Pullout work vs. fiber yield strength.

The pullout work is calculated as the area under the load vs. slip displacement curve. In Figure 13, both experimental and numerical values for fibers inclined at 30${}^{\circ }$ and 60${}^{\circ }$ are depicted. Note that as a general trend, the pullout work increases with the increase of fiber yield strength, and such a tendency is correctly captured by our numerical model.

## 5. Parametric Studies

In this section, the influences of both material properties (fiber yield strength, matrix tensile strength, interface bond) and geometric factors (fiber diameter, embedment length and inclination angle) on the overall pullout behavior, in particular on the maximum pullout load, are studied.

### 5.1. Interface-Related Parameters

The influence of the bond strength, the critical slip displacement, the internal frictional resistance and the friction coefficient is herein explored.

#### 5.1.1. Interfacial Bond Strength and Internal Frictional Resistance

**Figure 14:**
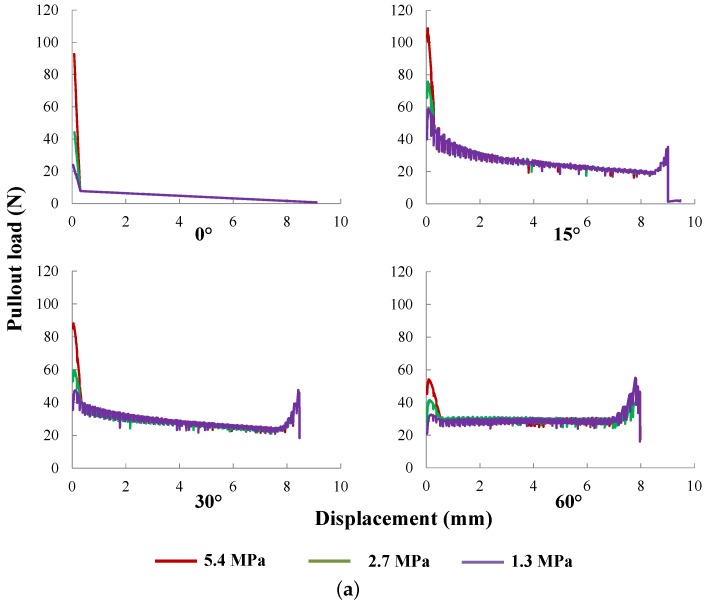

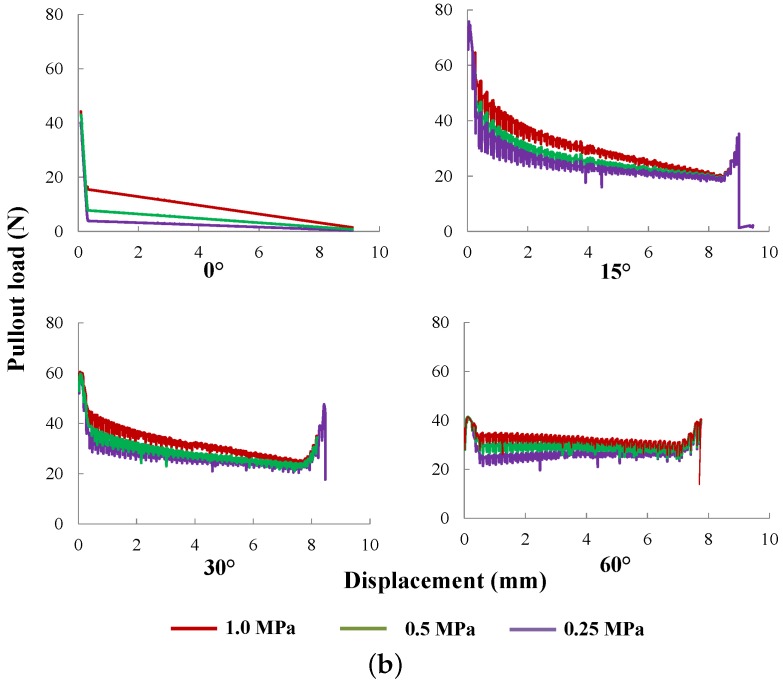
Pullout curves for: (**a**) $\tau_{max}$ at 1.3, 2.7 and 5.4 MPa; $\tau_{f_{c}}$ is kept at 0.5 MPa; and (**b**) $\tau_{f_{c}}$ at 0.25, 0.50 and 1.0 MPa, whereas $\tau_{max}$ is fixed at 2.7 MPa. Fibers are inclined at 0${}^{\circ }$, 15${}^{\circ }$, 30${}^{\circ }$ and 60${}^{\circ }$, respectively.

As described above, the interfacial bond between fiber and matrix is mainly composed of two parts, internal frictional resistance and bond strength. Simulations focusing on varying $\tau_{max}$ and $\tau_{f_{c}}$ separately for fibers inclined at 0${}^{\circ }$, 15${}^{\circ }$, 30${}^{\circ }$ and 60${}^{\circ }$ are carried out. The obtained pullout curves are demonstrated in Figure 14, where three different values, 1.3, 2.7 and 5.4 MPa for $\tau_{max}$, 0.25, 0.50 and 1.0 MPa for $\tau_{f_{c}}$, are considered. It is noted that $\tau_{max}$ has a dominant influence at the pre-peak stage (when the slip-end displacement is less than 0.3 mm), in particular over the peak load, whereas $\tau_{f_{c}}$ plays a more important role during the post-peak phase. In addition, the total slip displacement decreases as the inclination angle increases. This means the critical embedment length decreases with the inclination angle.

It needs to be pointed out that the highest peak load is obtained when the inclination angle is at 15${}^{\circ }$ for all cases. This is confirmed in Section 5.2.5, in Table 5, where $\theta_{max}$ falls between 12${}^{\circ }$ and 15${}^{\circ }$ for different values of fiber yield strength.

#### 5.1.2. Critical Slip for Interfacial Bond

**Figure 15:**
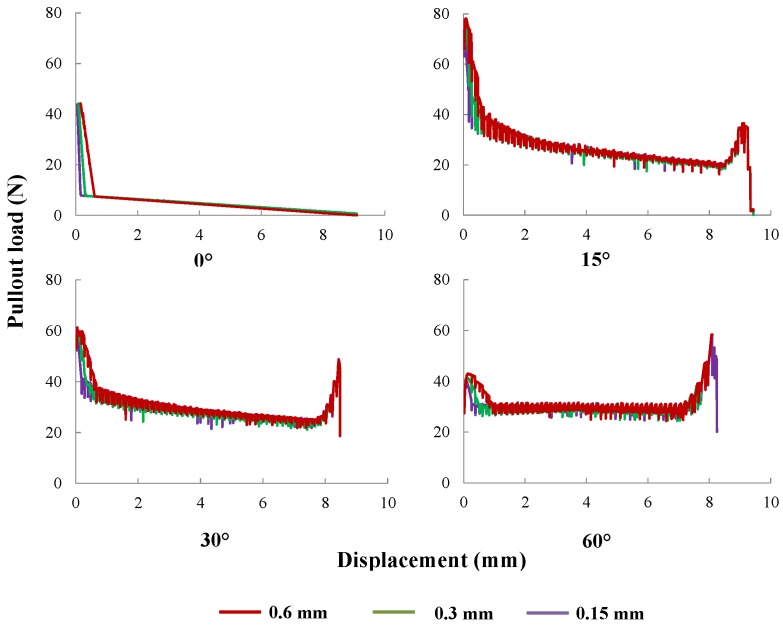
Pullout curves for $s_{b_{c}}$ equal to 0.15, 0.3 or 0.6 mm, with a fiber inclinations of 0${}^{\circ }$, 15${}^{\circ }$, 30${}^{\circ }$ and 60${}^{\circ }$, respectively.

As mentioned before, the critical slip displacement, $s_{b_{c}}$, denotes the maximum value for which interfacial bonding due to internal physical and chemical cohesion is active. Three different values, 0.15, 0.30 and 0.60 mm, of $s_{b_{c}}$ are selected to demonstrate such an effect for fibers inclined at 0${}^{\circ }$, 15${}^{\circ }$, 30${}^{\circ }$ and 60${}^{\circ }$, respectively, in Figure 15. Note that the difference of the pullout curves is only perceived at the first decaying branch; larger $s_{b_{c}}$ leads to a less steep drop from the maximum pullout load.

#### 5.1.3. Friction Coefficient

**Figure 16:**
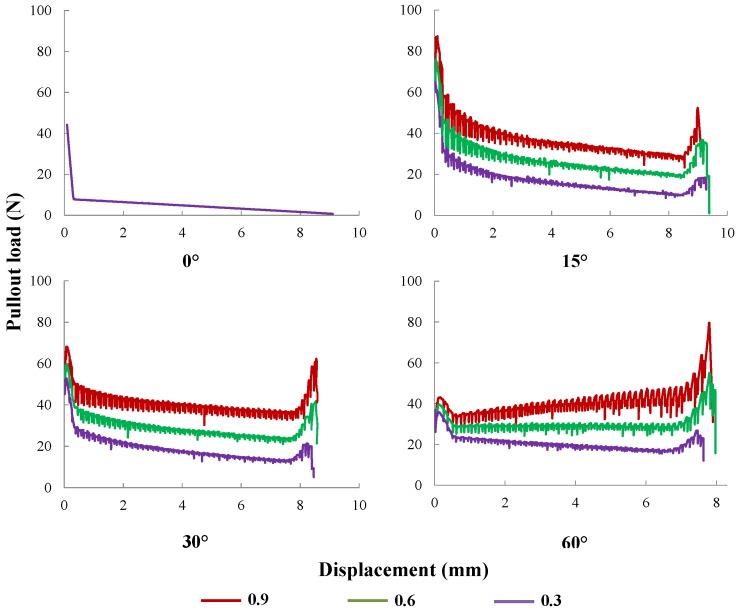
Pullout curves for *μ* equal to 0.3, 0.6 or 0.9, with fiber inclinations of 0${}^{\circ }$, 15${}^{\circ }$, 30${}^{\circ }$ and 60${}^{\circ }$, respectively.

As mentioned before, the interfacial friction consists of an internal friction and a pressure-dominated one due to fiber inclination (dry friction). The former is considered as a property of the interface, whereas the effect of the latter is only revealed when the fiber is inclined to the load direction. Four inclination angles (0${}^{\circ }$, 15${}^{\circ }$, 30${}^{\circ }$ and 60${}^{\circ }$) and three different values of the friction coefficient (0.3, 0.6 and 0.9) are simulated. The obtained pullout curves are demonstrated in Figure 16. Note that a higher friction coefficient leads to a load increment during the entire pullout process, and this effect is more pronounced for larger slip displacements. This evolution is attributable to the snubbing effect around the matrix cusp and corresponds well with the results of Cailleux [29].

### 5.2. Fiber-Related Parameters

Parameters related to fiber, such as the yield strength, fiber diameter and length and the inclination angle, all play an important role in the pullout curves. Herein, the influence of each parameter is studied while keeping the remaining ones unchanged.

#### 5.2.1. Fiber Yield Strength

**Figure 17:**
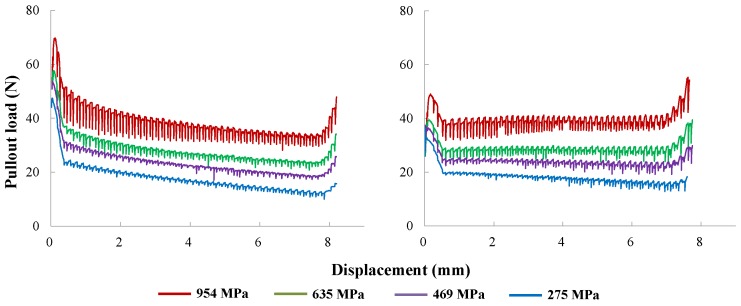
Pullout load vs. displacement result for fibers of different yield strengths at inclination angles of 30${}^{\circ }$ (**left**) and 60${}^{\circ }$ (**right**).

The influence of fiber yield strength has been experimentally demonstrated by Leung and Shapiro [26] and our numerical results in Figure 8. It is expected that when a fiber of the same size and surface treatment is pulled out, the same amount of energy will be consumed to overcome the interface bonding and frictional resistance. Furthermore, since the coefficient of friction is mainly dependent on the surface property, it is assumed constant for the same type of matrix and fiber. Consequently, the frictional energy will vary little with the fiber yielding strength. However, more energy is needed to bend the fiber as the yielding stress increases. This is clearly illustrated in Figure 17, where the entire pullout curves are moved upwards as the fiber yield strength is augmented. An interesting observation from Figure 17 is that the tail of the curve goes up, which was also seen in the experimental curves of Leung and Shapiro [26]. When the fiber is nearly pulled out, the short remaining end is rather stiff; thus, it may rotate as a short rigid cylinder, which subsequently cuts into the matrix. This has been explained previously through the first principal stress distribution in Figure 11, where the red areas represent the regions in heavy tension (nearly yielding), whereas the blue ones stand for the regions under less tension or compression.

#### 5.2.2. Fiber Diameter

**Figure 18:**
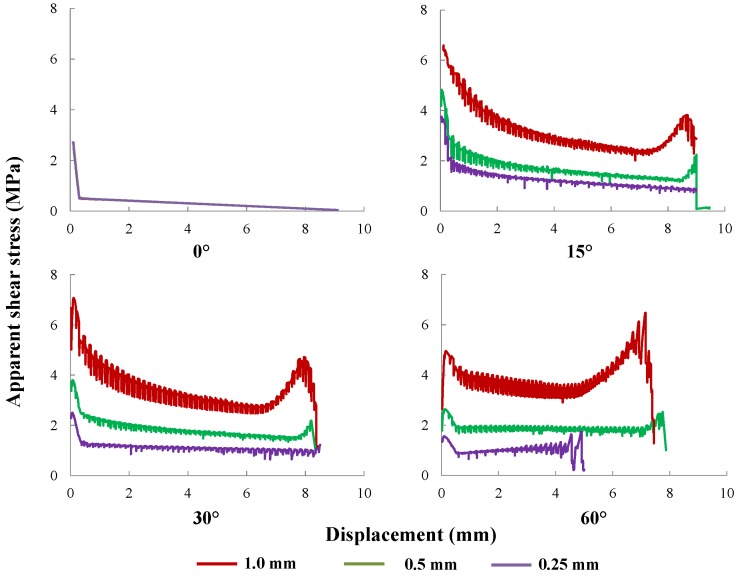
The influence of fiber diameter: apparent shear stress, $\tau^{*}$, vs. slip end displacement, *s*, for fibers of 1.0, 0.50 and 0.25 mm in diameter, inclined at 0${}^{\circ }$, 15${}^{\circ }$, 30${}^{\circ }$ and 60${}^{\circ }$, respectively.

The overall pullout response is closely related to the fiber diameter. First, a larger diameter will lead to a wider contact area between the fiber and the matrix. Consequently, a higher pullout load is attained. Second, different diameters lead to different moments of inertia of the fiber cross-section; thus, the dissipated energy will differ. To explore this influence in detail, three values of fiber diameter (0.25 mm, 0.50 mm, 1.00 mm) with four inclination angles (0${}^{\circ }$, 15${}^{\circ }$, 30${}^{\circ }$ and 60${}^{\circ }$) are selected to perform the numerical pullout simulations. The related parameters are documented in Table 4, whereas the pullout responses are shown in Figure 18. Additionally given in Table 4 is the aspect ratio, $L_{e}/d_{f}$, which is herein defined as the ratio between the fiber embedment length, $L_{e}$, and the fiber diameter, $d_{f}$. It is commonly accepted that the higher the aspect ratio is, the more efficient is the fiber at bridging cracks.

It is observed from Figure 18 that it is more difficult to pull a thicker fiber out. Consequently, more energy is consumed to pull an inclined thick fiber. Furthermore, the phenomenon of the rising tail of the pullout response is particularly noticeable for thicker fibers. In addition, the effect of bending is more pronounced for a thicker fiber, whereas more energy is consumed at the interface for a slim fiber. This explains the result that a larger peak pullout value is obtained at 30${}^{\circ }$ than at 15${}^{\circ }$ for the fiber of 1.0 mm in diameter, whereas the opposite is observed for the fibers of 0.25 mm and 0.5 mm in diameter. At the same time, it is noted that when a thin fiber is pulled, it may yield or get ruptured instead of being pulled out. This is the reason that the curve fluctuates around 5-mm-slip in the case of the 0.25 mm-diameter fiber inclined at 60${}^{\circ }$.

#### 5.2.3. Fiber Length

**Figure 19:**
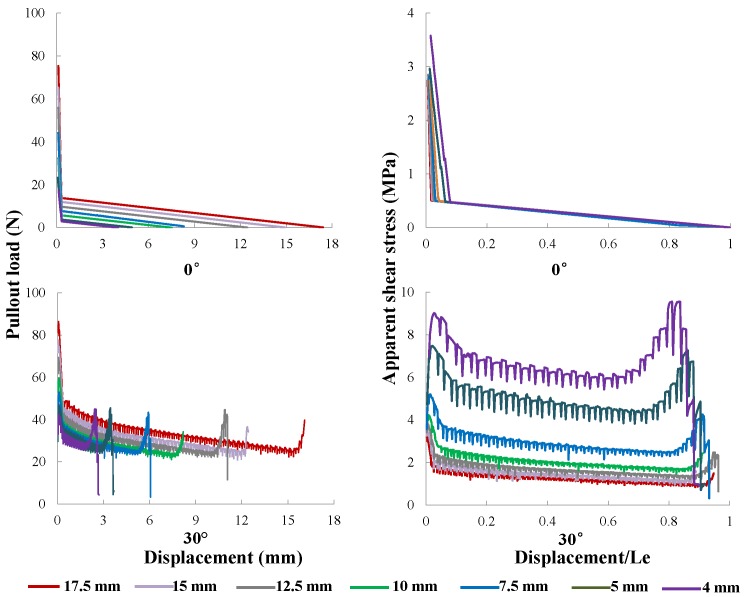
The influence of fiber length: pullout curves (**left**) and apparent bond stress vs. normalized slip displacement (**right**) for fibers inclined at (**top**) 0${}^{\circ }$ and (**bottom**) 30${}^{\circ }$, with $L_{e}$ varying from 4 to 17.5 mm, whereas the diameter is kept at 0.50 mm.

In Figure 19, the pullout responses for fibers of different lengths from 4 mm to 17.5 mm inclined at 0${}^{\circ }$ and 30${}^{\circ }$ are illustrated. Since a longer fiber length also means a wider interface area, thus a higher pullout load is attained. For a more clear representation, the apparent interfacial shear stress vs. relative slip normalized by the initial embedment length, which is equal to $L_{f}$, is also shown. It is observed that, for both aligned and inclined fibers, convergent results are achieved for longer fibers. This indicates that, when the rest is kept the same, the apparent bond strength coincides with the real one if the fiber embedment length is relatively large. Otherwise, the interface strength obtained through the pullout tests will be over-estimated.

#### 5.2.4. Fiber Inclination Angle

**Figure 20:**
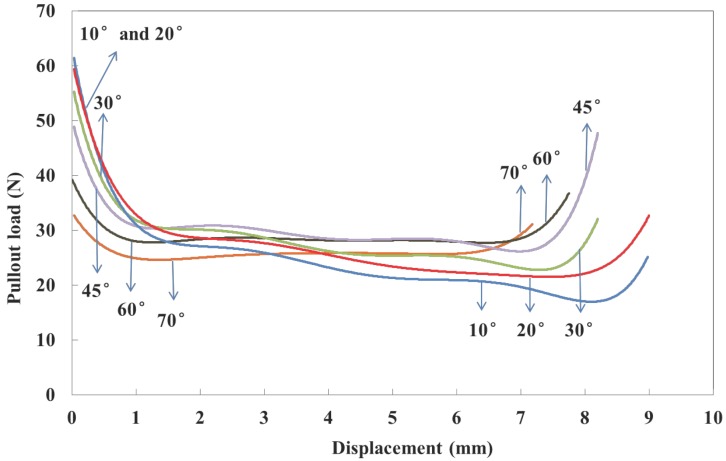
Numerical pullout load vs. slip displacement curves for inclination angles from 10${}^{\circ }$ to 70${}^{\circ }$.

The effect of the fiber inclination angle on the entire pullout response is explored for six orientations from 10${}^{\circ }$ to 70${}^{\circ }$ as demonstrated in Figure 20. Note that peak values for fibers inclined at 10${}^{\circ }$ and 20${}^{\circ }$ are higher than the rest, whereas the post-peak values during 2 to 7 mm only vary slightly. This is due to the fact that the pullout force is only a component of the bonding force before de-bonding. Afterwards, the contribution of fiber bending and friction is increased for all inclination angles.

#### 5.2.5. The Maximum Pullout Load

**Figure 21:**
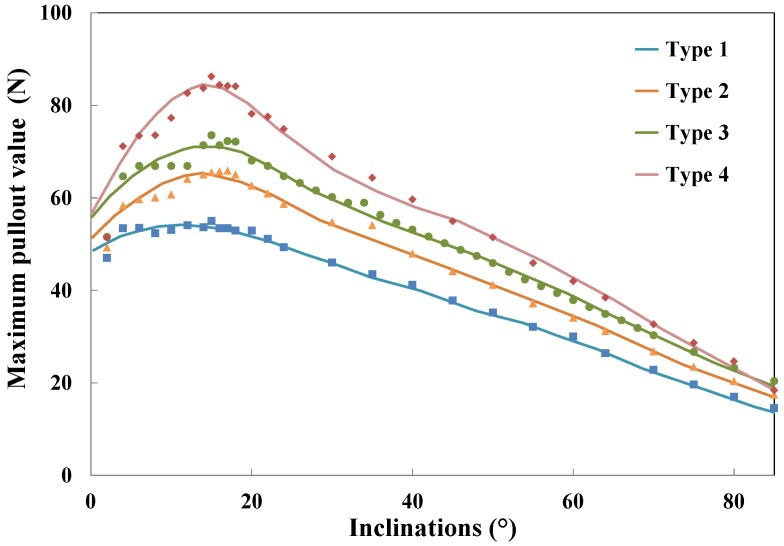
Maximum pullout load vs. inclination angle for the four yield strengths given in Table 2 and fitted curves using Equation (11) with parameters given in Table 5.

To explore the effect of fiber inclination, a spectrum of angles up to 85${}^{\circ }$ is simulated by keeping the fiber, matrix and interface properties constant. It is known that the length of the spalled matrix and the moment when the matrix spalls both matter in the pullout responses. According to Laranjeira et al. [35], spalling is considered to take place right after the beginning of fiber debonding, but prior to its full accomplishment. After some trial runs, this slip displacement is estimated, which is around 0.01 mm. The spalling length is calculated according to Laranjeira [35], which corresponds well with that of Leung and Shapiro [26]. Simulations of different fiber yield strengths are carried out, and the obtained pullout curves are plotted in Figure 21. The fitted function for the maximum pullout load in terms of fiber inclination angle is given by Equation (11), and the corresponding fitting parameters are listed in Table 5.

(11) $$\begin{array}{c} P_{max}\left(\theta \right)=F_{1}\;\exp\left[-{\left(\frac{\theta -\alpha_{1}}{\beta_{1}}\right)}^{2}\right]+F_{2}\;\exp\left[-{\left(\frac{\theta -\alpha_{2}}{\beta_{2}}\right)}^{2}\right] \end{array}$$

From Figure 21, when the fiber is inclined at $\theta_{max}$, which is around 15${}^{\circ }$, the maximum pullout load is the largest. After that, the maximum pullout load goes down almost linearly. This differs from the result of Morton and Groves [30] for a polyester resin matrix. This indicates that the optimum inclination angle for maximum pullout resistance varies with both fiber and matrix strength, as well as the interface properties.

### 5.3. Matrix-Related Parameter: The Tensile Strength

**Figure 22:**
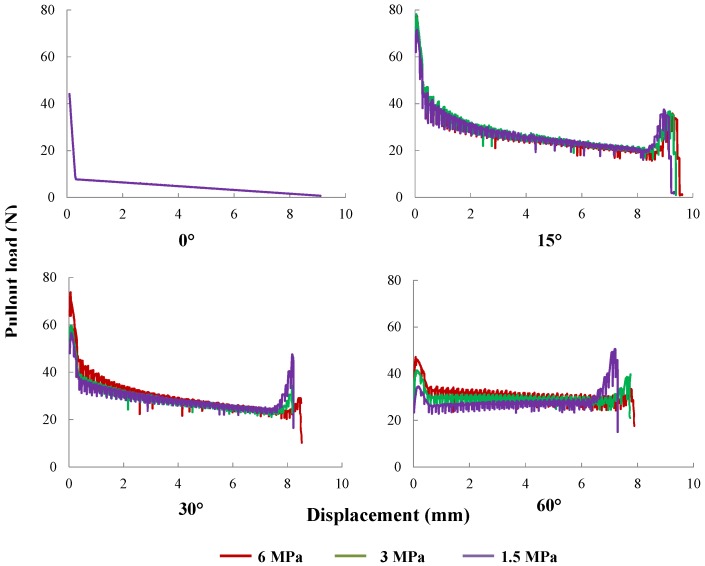
The influence of matrix tensile strength: pullout curves for matrix tensile strengths of 1.5 MPa, 3.0 MPa and 6.0 MPa; fibers inclined at 0${}^{\circ }$, 15${}^{\circ }$, 30${}^{\circ }$ and 60${}^{\circ }$, respectively.

The influence of matrix strength on the fiber pullout responses has been actively studied in the last three decades [24,38,39,40]. However, since the change in matrix strength may inevitably lead to slight [24] or marked [38,39,40] variation in the interface properties, the experimental observations can differ significantly. In the current numerical study, we isolate the matrix strength from the rest of material and geometric parameters to study its effect on the global pullout response. This is carried out through its direct influence on the matrix spalling length [35]. Taking the fiber with a yield strength of 635 MPa as an example, pullout out responses for three levels of matrix tensile strength (1.5, 3.0 and 6.0 MPa) and fiber inclined at 0${}^{\circ }$, 15${}^{\circ }$, 30${}^{\circ }$ and 60${}^{\circ }$ are explored. The corresponding spalling lengths calculated according to the work of Laranjeira et al. [35] are listed in Table 6. The obtained pullout curves are plotted in Figure 22. Note that a stronger matrix leads to a shorter spalling length and results in a higher peak load and a larger amount of absorption energy. However, a small difference is perceived for slip displacements between 3 mm and 7.5 mm for the fiber inclined at 30${}^{\circ }$, whereas more variations are observed for fibers inclined at 60${}^{\circ }$.

## 6. Conclusions

We have proposed a numerical model to explicitly reproduce the pullout behavior of a single fiber embedded within a cement-based matrix. This model takes into consideration the gradual deterioration of the interface bond, internal and dry friction, as well as matrix spalling. In particular, a constitutive law, which isolates the contributions of the internal bond, internal friction and dry friction is formed and validated. Cohesive elements endorsed with mixed-mode fracture capacities are implemented to represent the bond-slip behavior at the interface. Contact elements with Coulomb’s friction are placed at the interface to simulate frictional contact. Matrix spalling is modeled through material erosion to save computational time. The bond-slip behavior is first calibrated through pull-out curves for fibers aligned with the loading direction, then validated against experimental results carried out by Leung and Shapiro for steel fibers oriented at 30${}^{\circ }$ and 60${}^{\circ }$. The influence of fiber yield strength on the stress distribution within the fiber and the matrix, the effect of the inclination angle, as well as the matrix spalling length on the pullout response are all explored in detail.

The proposed methodology provides the necessary pull-out curves for a straight fiber oriented at a given angle for multi-scale models to study fracture in fiber-reinforced cementitious materials. The novelty lies in its capacity to capture the entire pullout process for a fiber with an arbitrary inclination angle. It is equally applicable for hooked or deformed fibers with increased computational efforts.

In addition, the following specific conclusions can be drawn from the parametric studies:

- Fiber critical length diminishes considerably for larger inclination angles.
- The matrix strength has a dominant influence at the initial stage of pullout, particularly on the maximum pullout load.
- The inclination angle at which the maximum peak pullout load is obtained differs when fiber, matrix and interface properties are different.

## Figures and Tables

**Table 1:** Material parameters for the matrix and the fiber given in [26].

	*ρ*	*E*	*ν*	$f_{c}$	$f_{t}$
	(kg/m^3^)	(GPa)	-	(MPa)	(MPa)
Matrix	2100	30	0.20	36.5 ± 2.5	$f_{c}/12$ (estimated)
Steel fiber	7800	200	0.33	-	-

**Table 2:** Yield and tensile strength of the four types of fibers tested in [26]; the corresponding $L_{c}$ is listed for a diameter of 0.5 mm, where ${}^{\left(1\right)}$ and ${}^{\left(2\right)}$ are calculated using the tensile strength, $f_{r}$, and the yield strength, $f_{y}$, respectively.

Fiber Type	1	2	3	4
$f_{y}$ (MPa)	275	469	635	954
$f_{r}$ (MPa)	-	783	847	1023
${}^{\left(1\right)}$$L_{c}$ (mm)	12.7	21.7	29.4	44.2
${}^{\left(2\right)}$$L_{c}$ (mm)	-	36.3	39.2	47.4

**Table 3:** Extracted parameters of the fiber-matrix interface from the experimental data of Leung and Shapiro [26].

$\tau_{\max}$	$\tau_{f_{c}}$	$s_{b_{c}}$	*μ*
(MPa)	(MPa)	(mm)	-
2.7 ± 0.1	0.5 ± 0.1	0.3	0.60

**Table 4:** Parameters for the case studies on fiber diameter and fiber length.

$L_{e}$	$d_{f}$	$L_{e}/d_{f}$	$T_{f}$	$H_{f}$	$\tau_{\max}$	$f_{y}$	$L_{c}$
(mm)	(mm)	-	(mm)	(mm)	(MPa)	(MPa)	(mm)
10	0.50	20	0.79	0.36	2.7	635	29.4
10	0.25	40	0.39	0.18	2.7	635	14.7
10	1.00	10	1.57	0.72	2.7	635	58.8
4.0	0.50	8	0.79	0.36	2.7	635	29.4
5.0	0.50	10	0.79	0.36	2.7	635	29.4
7.5	0.50	15	0.79	0.36	2.7	635	29.4
10.0	0.50	20	0.79	0.36	2.7	635	29.4
12.5	0.50	25	0.79	0.36	2.7	635	29.4
15.0	0.50	30	0.79	0.36	2.7	635	29.4
17.5	0.50	35	0.79	0.36	2.7	635	29.4

**Table 5:** Fitted parameters for the maximum pullout load vs. fiber inclination angle, where $\theta_{max}$ is the angle where the maximum peak load is attained.

Fiber Type	$F_{1}$	$F_{2}$	$\alpha_{1}$	$\alpha_{2}$	$\beta_{1}$	$\beta_{2}$	$\theta_{\max}$
	(N)	(N)	(${}^{\circ }$)	(${}^{\circ }$)	(${}^{\circ }$)	(${}^{\circ }$)	(${}^{\circ }$)
1	15.5	42.0	7.9	26.2	19.7	56.4	12.4
2	18.1	49.4	12.7	26.7	16.2	55.7	15.3
3	20.1	54.2	12.6	27.4	16.0	55.9	15.2
4	33.7	59.0	12.7	33.0	14.6	48.2	15.3

**Table 6:** Spalling length for three levels of matrix tensile strength and fiber at four different inclination angles.

$f_{t}$ (MPa)	1.5	3.0	6.0
	**Spalling**	**Length**	**in mm**
0${}^{\circ }$	0.00	0.00	0.00
15${}^{\circ }$	0.59	0.41	0.27
30${}^{\circ }$	1.50	1.03	0.71
60${}^{\circ }$	3.31	2.31	1.60

## Abbreviations

The following abbreviations are used in this manuscript:

$A_{f}$	Fiber cross-section area
$d_{f}$	Fiber diameter
*E*	Elastic modulus
$f_{c}$	Matrix compressive strength
$f_{r}$	Fiber tensile strength
$f_{t}$	Matrix tensile strength
$f_{y}$	Fiber yield strength
$H_{f}$	Modeled fiber height
$L_{c}$	Critical fiber (embedment) length
$L_{e}$	Fiber embedment length
$L_{f}$	Fiber length
$L_{sp}$	Matrix spalling length
$P_{max}$	Maximum pullout load
$P_{f}$	Frictional peak load
p(θ)	Compressive stress at an inclination angle of *θ*
*s*	Slip displacement
$s_{b_{c}}$	Critical slip for interfacial bond
$T_{f}$	Modeled fiber thickness (out-of-plane dimension)
*μ*	Coulomb’s friction coefficient
*ν*	Poisson’s ratio
*ρ*	Material density
*θ*	Fiber inclination angle with respect to the load direction
$\tau^{*}$	Apparent interfacial shear stress
τ(s,θ)	Interfacial shear stress as a function of *s* and *θ*
$\tau_{b}\left(s\right)$	Internal bonding as a function of *s*
$\tau_{f_{c}}$	Internal frictional resistance
$\tau_{max}$	Interfacial shear strength (maximum shear stress)
